# Authentication of variable length messages in quantum key distribution

**DOI:** 10.1140/epjqt/s40507-022-00127-0

**Published:** 2022-02-16

**Authors:** Khodakhast Bibak, Bruce M. Kapron, Venkatesh Srinivasan

**Affiliations:** 1 Department of Computer Science and Software Engineering, Miami University, Oxford, Ohio 45056 USA; 2 Department of Computer Science, University of Victoria, Victoria, BC V8W 3P6 Canada

**Keywords:** Quantum key distribution, Polynomial Hash, *ε*-almost-strongly universal, Polynomial congruence

## Abstract

Authentication plays a critical role in the security of quantum key distribution (QKD) protocols. We propose using Polynomial Hash and its variants for authentication of variable length messages in QKD protocols. Since universal hashing is used not only for authentication in QKD but also in other steps in QKD like error correction and privacy amplification, and also in several other areas of quantum cryptography, Polynomial Hash and its variants as the most efficient universal hash function families can be used in these important steps and areas, as well. We introduce and analyze several efficient variants of Polynomial Hash and, using deep results from number theory, prove that each variant gives an *ε*-almost-Δ-universal family of hash functions. We also give a general method for transforming any such family to an *ε*-almost-strongly universal family of hash functions. The latter families can then, among other applications, be used in the Wegman–Carter MAC construction which has been shown to provide a universally composable authentication method in QKD protocols. As Polynomial Hash has found many applications, our constructions and results are potentially of interest in various areas.

## Introduction

Key establishment protocols, in which cryptographic keys are securely exchanged between parties over a public channel, usually use methods from public-key cryptography, like Diffie–Hellman key exchange (DH) and elliptic-curve Diffie–Hellman (ECDH); see [1] for a comprehensive treatment of the key establishment protocols in cryptography. However, the security of such schemes relies on the computational hardness of certain mathematical problems (namely, the discrete logarithm problem, the elliptic-curve discrete logarithm problem, and the integer factorization problem) which can be solved on a sufficiently powerful quantum computer running Shor’s algorithm. Quantum key distribution (QKD), which relies on the foundations of quantum mechanics, provides a higher level of security than such schemes. QKD is provably secure even against an adversary with unbounded computational power and is also becoming increasingly feasible to implement. QKD has found many surprising applications, its commercialization has been successful, and QKD networks are now deployed in some metropolitan areas [2]. There are many excellent surveys on QKD (see, e.g., [3–5]).

Studying the security of QKD has become a topic of great importance (see [6, 7] for two excellent surveys). QKD requires a quantum channel and a classical channel. The classical channel needs to be authenticated to avoid man-in-the-middle (MITM) attacks. For the authentication of the communications on the classical channel, the original message authentication codes (MACs) proposed by Wegman and Carter [8], its variants [9], or other efficient constructions [10] are used. All these MACs use universal hash functions in their constructions. In the Wegman–Carter paradigm [8] the message is first hashed with an *ε*-almost-strongly universal hash function and then encrypted with a one-time pad. The application of the Wegman–Carter paradigm in QKD was originally proposed by Bennett and Brassard [11, 12] in the BB84 protocol (their well-known QKD scheme developed in 1984) and by Bennett et. al. [13], and since then has been studied extensively (see, e.g., [9, 10, 14–18]). The Wegman–Carter MAC construction is described as follows. The legitimate parties share a secret hash function chosen uniformly at random from an *ε*-almost-strongly universal (*ε*-ASU) family of hash functions, and a secret encryption key (a sequence of random one-time pads). A message is authenticated by first hashing it with the shared hash function and then encrypting the resulting hash value with the shared encryption key (shared one-time pad). The resulting encrypted hash value, called an *authentication tag*, is transmitted together with the message (as a pair). Upon receiving this pair, the legitimate party recomputes and validates it. Such a MAC algorithm is *information-theoretically* (*unconditionally*) secure, that is, even an adversary who has unbounded computational power cannot forge the MAC with probability greater than the collision probability of the hash function family [8].

Because, in the authentication of the classical channel, the legitimate parties need to share some initial small secret information in advance as described above, QKD is sometimes called a quantum key *growing* (rather than quantum key distribution) protocol. The Wegman–Carter MAC construction has been shown in [18] to be universally composable (UC) [19–21], and therefore it is sufficient for authentication in QKD systems. One way to make QKD protocols more efficient and applicable is to construct efficient *ε*-ASU hash function families because these families are the main ingredient in the Wegman–Carter construction (and in many other universal hashing based MACs).

In this paper, following [22–24] we propose using *Polynomial Hash* (PH) and its variants for authentication of *variable length messages* in QKD systems. Since universal hashing is used not only for authentication in QKD but also in other steps in QKD like error correction and privacy amplification [7, 13, 25–30], and also in several other areas of quantum cryptography (that we will briefly mention in the last section), Polynomial Hash and its variants as the most efficient universal hash function families can be used in these important steps and areas, as well. Polynomial Hash is a well-known *ε-almost-*Δ*-universal* (*ε*-AΔU) family of hash functions which has found various important applications, for example, Galois/Counter Mode (GCM) [31] (which is used in IPsec, SSH, and TLS) and Poly1305 [32] (which is used in Google Chrome’s TLS, and later was added to OpenSSH) use this scheme. See also [33–43] for various other applications of Polynomial Hash. We introduce and analyze several efficient variants of Polynomial Hash and, using deep results from number theory, prove that such variants are also *ε*-AΔU and so can be used in various applications. Furthermore, we propose a general method by which any *ε*-AΔU hash function family can be transformed to an *ε*-ASU family. Therefore, the Polynomial Hash variants constructed in this paper can all be transformed to *ε*-ASU families which makes them useful for various applications including authentication of variable length messages in QKD.

The rest of this paper is organized as follows. In Sect. 2, we review some results on equations over fields and rings, in particular some rather underappreciated results of Konyagin [44, 45], using which we obtain upper bounds for the number of solutions of polynomial congruences over the ring of integers modulo *n*, $\mathbb{Z}_{n}$. In Sect. 3, we formally define universal hashing and its variants and prove a general result for transforming *ε*-AΔU families to *ε*-ASU families. In Sect. 4, we construct and analyze several efficient variants of Polynomial Hash and compare our results with available results.

## Equations over fields and rings

Throughout the paper, *n* is a positive integer, *p* is a prime, $\mathbb{Z}_{n}$ is the ring of integers modulo *n* defined as $\mathbb{Z}_{n} = \lbrace 0, \ldots , n-1 \rbrace $, $\mathbb{F}$ is a field, and $\mathbb{F}_{q}$ is the finite field with *q* elements, where *q* is a prime power. Also, $\mathbb{F}_{p}$ is the prime field. Note that $\mathbb{F}_{p}=\mathbb{Z}_{p}=\lbrace 0, \ldots , p-1 \rbrace $.

Finding (the number of) solutions of univariate and multivariate polynomial equations over fields and rings is a fundamental problem in mathematics, computer science, and related areas with many applications in various domains. In this paper, by a polynomial we mean a univariate polynomial. As a classical example, one can mention the Fundamental Theorem of Algebra which gives the exact number of solutions of polynomial equations over the field of complex numbers.

### Theorem 2.1

(Fundamental Theorem of Algebra)

*Let* $f(x)$ *be a non*-*zero polynomial of degree* $d \geq 0$ *with complex coefficients*. *Then the equation*

$$ f(x) = 0$$

*has*, *counting multiplicities*, *exactly* *d* *complex solutions*. *Equivalently*, *the field of complex numbers is algebraically closed*.

There are about a hundred proofs(!) of the Fundamental Theorem of Algebra [46]. See [46] for “one of the most elegant and certainly the shortest” proof.

By a *solution* of the polynomial congruence

$$ f(x) \equiv 0 \ (\operatorname {mod} n)$$

we mean an integer in $\mathbb{Z}_{n}$ that satisfies the congruence. So, every polynomial congruence modulo *n* has at most *n* solutions. Similarly, every multivariate polynomial congruence in *k* variables modulo *n* has at most $n^{k}$ solutions.

A natural question is whether the Fundamental Theorem of Algebra can be applied to the ring $\mathbb{Z}_{n}$ (that is, to polynomial congruences modulo *n*)? The answer is *no*; there is no direct analog of the Fundamental Theorem of Algebra for polynomial congruences. Let us see some examples. The following result, proved by D. N. Lehmer [47], gives an explicit formula for the number of solutions of linear congruences:

### Theorem 2.2

(Lehmer’s Theorem)

*Let* $a_{1},\ldots ,a_{k},b\in \mathbb{Z}$. *The linear congruence*

$$ a_{1}x_{1}+\cdots +a_{k}x_{k}\equiv b \ (\operatorname {mod} n)$$

*has a solution* $\langle x_{1},\ldots ,x_{k} \rangle \in \mathbb{Z}_{n}^{k}$ *if and only if* $\ell \mid b$, *where* $\ell =\gcd (a_{1}, \ldots , a_{k}, n)$. *Furthermore*, *if this condition is satisfied*, *then there are* $\ell n^{k-1}$ *solutions*.

Note that the generalization of Lehmer’s Theorem to higher degree multivariate polynomial congruences is a challenging problem. In fact, even the quadratic version addressed by Cohen [48] has much more complicated formulas.

By Lehmer’s Theorem, the linear congruence $ax \equiv b \ (\operatorname {mod} n)$, where *a* and *b* are integers, has zero, one, or more solutions (in fact, zero or $\gcd (a,n)$ solutions). As another example, the quadratic congruence $x^{2} \equiv 1 \ (\operatorname {mod} 8)$ has four solutions 1, 3, 5, and 7. These examples show that the Fundamental Theorem of Algebra is not applicable to polynomial congruences. But when the modulus is *prime*, we have the following result due to Lagrange which gives *an upper bound* for the number of solutions (see, e.g., [49]).

### Theorem 2.3

(Lagrange’s Theorem)

*Given a prime* *p*, *let*

$$ f(x)=a_{d}x^{d} + \cdots + a_{1}x + a_{0} $$

*be a polynomial with integer coefficients such that* $a_{d} \not \equiv 0 \ (\operatorname {mod} p)$ (*said to be of degree* *d*). *Then the polynomial congruence*

$$ f(x) \equiv 0 \ (\operatorname {mod} p)$$

*has at most* *d* *solutions*.

Lagrange’s Theorem can be extended from the prime field $\mathbb{Z}_{p}$ to arbitrary fields (not necessarily finite) as the following (see, e.g., [50]):

### Theorem 2.4

*Let* $\mathbb{F}$ *be a field and* $f(x)$ *be a non*-*zero polynomial of degree* $d \geq 0$ *with coefficients in* $\mathbb{F}$. *Then the polynomial equation*

$$f(x) = 0 $$

*has*, *counting multiplicities*, *at most* *d* *solutions in* $\mathbb{F}$. *Therefore*, *it has at most* *d* *distinct solutions in* $\mathbb{F}$.

It would be useful to compare the above results:

### Remark 2.5

The following observations are useful, specially when discussing the Polynomial Hash and its variants:

- Setting $\mathbb{F}=\mathbb{Z}_{p}$ in Theorem 2.4 we obtain Lagrange’s Theorem but not in full generality. In fact, in Theorem 2.4 when $\mathbb{F}=\mathbb{Z}_{p}$, the coefficients of the polynomial must be in $\mathbb{Z}_{p}$, but in Lagrange’s Theorem the coefficients are arbitrary integers.
- While Theorem 2.4 works on arbitrary fields (including the field of complex numbers), it does not imply the Fundamental Theorem of Algebra. In fact, the Fundamental Theorem of Algebra gives the *exact* number of complex solutions of polynomial equations over the field of complex numbers, but Lagrange’s Theorem and Theorem 2.4 just give *upper bounds* for the number of solutions over the prime field and arbitrary fields, respectively.
- The proof of the Fundamental Theorem of Algebra is totally different from the proof of Lagrange’s Theorem and Theorem 2.4. In fact, the proof of the Fundamental Theorem of Algebra is usually given as a result in complex analysis and “the shortest” proof [46] still requires two pages, but the proofs of Lagrange’s Theorem and Theorem 2.4 are usually given as results in number theory and field theory and can be written in just a few lines (see, e.g., [49, 50]).

Note that Lagrange’s Theorem does not hold for composite moduli. For example, the quadratic congruence $x^{2} \equiv 1 \ (\operatorname {mod} 8)$ has four solutions 1, 3, 5, and 7. Surprisingly, Vandiver [51] obtained, for ‘restricted’ solutions, exactly the same upper bound as in Lagrange’s Theorem and Theorem 2.4 in the much more general setting of commutative rings with identity (that we call Vandiver’s Theorem), but, unfortunately, his result, while is quite interesting, seems to have been forgotten. Let ${\mathcal{R}}$ be a commutative ring with identity. Two elements $u, v \in {\mathcal{R}}$ are said to be *absolutely distinct* if $u-v$ is not zero and not a zero divisor.

### Theorem 2.6

(Vandiver’s Theorem)

*Let* ${\mathcal{R}}$ *be a commutative ring with identity*. *Let*

$$ f(x)=a_{d}x^{d} + \cdots + a_{1}x + a_{0} $$

*be a polynomial with coefficients in* ${\mathcal{R}}$ *such that* $a_{d} \neq0$. *Then the polynomial equation*

$$ f(x) = 0$$

*has at most* *d* *absolutely distinct solutions*.

Taking ${\mathcal{R}}=\mathbb{Z}_{n}$, Vandiver [51] derived the following version for $\mathbb{Z}_{n}$. Two integers *a* and *b* are said to be *absolutely incongruent* modulo *n* if $a-b$ is coprime to *n*.

### Theorem 2.7

(Vandiver’s Theorem for $\mathbb{Z}_{n}$)

*Given a positive integer* *n*, *let*

$$ f(x)=a_{d}x^{d} + \cdots + a_{1}x + a_{0} $$

*be a polynomial with integer coefficients such that* $a_{d} \not \equiv 0 \ (\operatorname {mod} n)$. *Then the polynomial congruence*

$$ f(x) \equiv 0 \ (\operatorname {mod} n)$$

*has at most* *d* *absolutely incongruent solutions*.

Note that setting $n=p$, a prime, in Vandiver’s Theorem for $\mathbb{Z}_{n}$, we re-obtain Lagrange’s Theorem since any two distinct elements of $\mathbb{Z}_{p}$ are absolutely incongruent modulo *p*.

The rest of this section is devoted to generalizing Lagrange’s Theorem to composite moduli (or, equivalently, generalizing Vandiver’s Theorem for $\mathbb{Z}_{n}$ to cover *all* solutions). For generalization to prime power moduli, an upper bound for the number of solutions can be obtained using the following result (see, e.g., [49]).

### Theorem 2.8

*Suppose* $\alpha > 1$ *is an integer and* *s* *is a solution of the polynomial congruence*

$$ f(x) \equiv 0 \ (\operatorname {mod} p^{\alpha -1}).$$

*Then we have the following cases*:

- *If* $f'(s) \not \equiv 0 \ (\operatorname {mod} p)$ *then* *s* *can be lifted in a unique way from* $p^{\alpha -1}$ *to* $p^{\alpha }$. *That is*, *there is a unique* $t \in \mathbb{Z}_{p^{\alpha }}$ *which generates* *s* *and which satisfies the polynomial congruence*
  $$ f(x) \equiv 0 \ (\operatorname {mod} p^{\alpha}).$$
- *If* $f'(s) \equiv 0 \ (\operatorname {mod} p)$ *then*:
  - *If* $f(s) \equiv 0 \ (\operatorname {mod} p^{\alpha})$, *s* *can be lifted from* $p^{\alpha -1}$ *to* $p^{\alpha }$ *in* *p* *distinct ways*.
  - *If* $f(s) \not \equiv 0 \ (\operatorname {mod} p^{\alpha})$, *s* *cannot be lifted from* $p^{\alpha -1}$ *to* $p^{\alpha }$.

Given a positive integer *n*, let

$$ f(x)=a_{d}x^{d} + \cdots + a_{1}x + a_{0} $$

be a polynomial with integer coefficients such that $a_{d} \not \equiv 0 \ (\operatorname {mod} n)$ (said to be of degree *d*). Denote by $N_{d}(a_{0}, a_{1}, \ldots , a_{d}, n)$ the number of solutions of the polynomial congruence

$$ f(x) \equiv 0 \ (\operatorname {mod} n).$$

### Lemma 2.9

*If* $\mathcal{G}:=\gcd (a_{0}, a_{1}, \ldots , a_{d}, n)>1$ *then*

$$ N_{d}(a_{0}, a_{1}, \ldots , a_{d}, n)=\mathcal{G}N_{d}(a_{0}/ \mathcal{G}, a_{1}/ \mathcal{G}, \ldots , a_{d}/\mathcal{G}, n/ \mathcal{G}). $$

### Proof

The proof easily follows from the basic properties of congruences. □

Therefore, by Lemma 2.9, it suffices to consider the number of solutions of the above polynomial congruence with $\gcd (a_{0}, a_{1}, \ldots , a_{d}, n)=1$. For simplicity, we denote the number of such solutions by $N(d, n)$.

Using Lagrange’s Theorem and Theorem 2.8, we can obtain the following upper bound for $N(d, p^{\alpha })$.

### Theorem 2.10

*Let* $\alpha \geq 1$ *be an integer*. *Then*

$$ N\bigl(d, p^{\alpha }\bigr) \leq dp^{\alpha -1}.$$

### Proof

Clearly, if $N(d, p)=0$ then $N(d, p^{\alpha })=0$, for all integers $\alpha \geq 1$. So, let $N(d, p)>0$. Then, using Theorem 2.8, corresponding to each solution of the polynomial congruence modulo *p* there will be 0, 1, or *p* solutions modulo $p^{2}$. So, using Lagrange’s Theorem and Theorem 2.8, $N(d, p^{2}) \leq dp$. Similarly, corresponding to each solution of the polynomial congruence modulo $p^{2}$ there will be 0, 1, or *p* solutions modulo $p^{3}$. Therefore, $N(d, p^{3}) \leq dp^{2}$. Repeating this process, the result follows. □

Is there a better upper bound for $N(d, p^{\alpha })$? Yes(!), and the best upper bound for $N(d, p^{\alpha })$ is widely attributed to Stewart [52], and to Schmidt and Stewart [53]. But we have discovered that Konyagin [44, 45] (in Russian and back in 1979) has already obtained a stronger and more general upper bound for $N(d, p^{\alpha })$ (that we call Konyagin’s Theorem). We remark that all these bounds were obtained using advanced tools in number theory and their proofs are rather long and complicated.

### Theorem 2.11

(Konyagin’s Theorem)

*Let* $\alpha \geq 1$ *be an integer*. *Then*

$$ N\bigl(d, p^{\alpha }\bigr) \leq \frac{d}{\alpha (p-1)}p^{\alpha }.$$

*Furthermore*, *if* $d \geq 2$ *and* $p \geq d^{1+1/(d-1)}$, *then*

$$ N\bigl(d, p^{\alpha }\bigr) \leq p^{\alpha (1-1/d)}.$$

So far, we have very good upper bounds for the number of solutions of polynomial congruences modulo prime powers. Now, we generalize these upper bounds to arbitrary moduli. For this we need the following tool (see, e.g., [49]).

### Theorem 2.12

*Let* $f(x)$ *be a polynomial with integer coefficients*. *Also*, *let* $n_{1},\ldots ,n_{r}$ *be positive integers*, *pairwise coprime*, *and let* $n = n_{1} \cdots n_{r}$. *Then the polynomial congruence*

1 $$\begin{aligned} f(x) \equiv 0 \ (\operatorname {mod} n) \end{aligned}$$

*has a solution if and only if each of the polynomial congruences*

2 $$\begin{aligned} f(x) \equiv 0 \ (\operatorname {mod} n_{i}) \quad (i = 1, \dots , r) \end{aligned}$$

*has a solution*. *Moreover*, *if* $v(n)$ *and* $v(n_{i})$ *denote the number of solutions of* (1) *and* (2), *respectively*, *then*

$$ v(n) = v(n_{1}) \cdots v(n_{r}). $$

When modulus *n* is square-free, we obtain the best upper bound for $N(d, n)$ using Lagrange’s Theorem and Theorem 2.12 as follows.

### Theorem 2.13

*Let* *n* *be square*-*free with* *r* *distinct prime factors*. *Then*

$$ N(d, n) \leq d^{r}.$$

### Proof

Let *n* has the prime factorization $n= p_{1} \ldots p_{r}$, where $p_{i}$’s are distinct primes. By Lagrange’s Theorem, $N(d, p_{i}) \leq d$ for all *i*. Since $p_{i}$’s are pairwise coprime, using Theorem 2.12 we have

$$ N(d, n) = \prod_{i=1}^{r}N(d, p_{i}) \leq d^{r}.$$

 □

Similarly, when modulus *n* is an arbitrary positive integer, we obtain the best upper bound for $N(d, n)$ using Konyagin’s Theorem and Theorem 2.12 as follows.

### Theorem 2.14

*Let* $n>1$ *has the prime factorization* $n= \prod_{i=1}^{r}p_{i}^{\alpha _{i}}$, *where* $p_{i}$*’s are prime and* $\alpha _{i} \geq 1$ *for all* *i*. *Then*

$$ N(d, n) \leq \frac{nd^{r}}{\prod_{i=1}^{r}\alpha _{i}(p_{i}-1)}.$$

*Furthermore*, *if* $d \geq 2$ *and* $p_{i} \geq d^{1+1/(d-1)}$ *for all* *i*, *then*

$$ N(d, n) \leq \frac{n}{\prod_{i=1}^{r} p_{i}^{\alpha _{i}/d}}.$$

### Proof

By Konyagin’s Theorem, we have

$$ N\bigl(d, p_{i}^{\alpha _{i}}\bigr) \leq \frac{d}{\alpha _{i}(p_{i}-1)}p_{i}^{ \alpha _{i}},$$

for all *i*. Since $p_{i}^{\alpha _{i}}$’s are pairwise coprime, using Theorem 2.12 we have

$$ N(d, n) = \prod_{i=1}^{r}N\bigl(d, p_{i}^{\alpha _{i}}\bigr) \leq \prod_{i=1}^{r} \frac{d}{\alpha _{i}(p_{i}-1)}p_{i}^{\alpha _{i}}= \frac{nd^{r}}{\prod_{i=1}^{r}\alpha _{i}(p_{i}-1)}.$$

Similarly, if $d \geq 2$ and $p_{i} \geq d^{1+1/(d-1)}$ for all *i*, then by Konyagin’s Theorem and Theorem 2.12, we have

$$ N(d, n) = \prod_{i=1}^{r}N\bigl(d, p_{i}^{\alpha _{i}}\bigr) \leq \prod_{i=1}^{r}p_{i}^{ \alpha _{i}(1-1/d)}= \frac{n}{\prod_{i=1}^{r} p_{i}^{\alpha _{i}/d}}.$$

 □

## Universal hashing and its variants

Universal hash function families, introduced by Carter and Wegman [54], guarantee a low number of collisions in expectation when a hash function is chosen uniformly at random from the universal hash function family. These hash function families have many important applications in computer science and cryptography (see [55] for a comprehensive list of references). We begin by describing universal hashing and its variants in detail [54, 56–60]. For a set $\mathcal{X}$, we write $x \leftarrow \mathcal{X}$ to denote that *x* is chosen uniformly at random from $\mathcal{X}$.

### Definition 3.1

Let *H* be a family of functions from a finite domain *D* to a finite range *R*, and let *ε* be a constant such that $\frac{1}{|R|} \leq \varepsilon < 1$.

- The family *H* is a *universal* family of hash function if the probability, over a random choice of a hash function from *H*, that two distinct elements of *D* *collide* (i.e., have the same hash value) is at most $1/|R|$ (that is, distinct elements of *D* do not collide too often). Formally, *H* is universal if for any two distinct $x,y\in D$, we have $\operatorname{Pr}_{h \leftarrow H}[h(x) = h(y)] \leq \frac{1}{|R|}$. Also, *H* is an *ε-almost universal* (*ε*-AU) family of hash functions if for any two distinct $x,y\in D$, we have $\operatorname{Pr}_{h \leftarrow H}[h(x) = h(y)] \leq \varepsilon $. Note that an *ε*-AU family, for a sufficiently small *ε*, is *close* to being universal.
- Suppose *R* is a finite additive Abelian group. The family *H* is a Δ-*universal* family of hash functions if, given a randomly chosen hash function from *H*, the difference of the hash values of any two distinct elements of *D* is uniformly distributed in *R*. Formally, *H* is Δ-universal if for any two distinct $x,y\in D$, and all $b\in R$, we have $\operatorname{Pr}_{h \leftarrow H}[h(x) - h(y) = b] = \frac{1}{|R|}$, where ‘−’ denotes the group subtraction operation. Also, *H* is an *ε-almost-*Δ*-universal* (*ε*-AΔU) family of hash functions if for any two distinct $x,y\in D$, and all $b\in R$, we have $\operatorname{Pr}_{h \leftarrow H}[h(x) - h(y) = b] \leq \varepsilon $. When $R=\mathbb{Z}_{2}^{k}=\{0,1\}^{k}$ for some *k*, the operation ‘−’ can be replaced by ‘⊕’ (XOR), and *H* is also called *ε-almost XOR universal* (*ε*-AXU) or *ε-otp-secure*.
- The family *H* is a *strongly universal* (or 2*-wise-independent*) family of hash functions if, given a randomly chosen hash function from *H*, the hash values of any two distinct elements of *D* are independent and uniformly distributed in *R*. Formally, *H* is strongly universal if for any two distinct $x,y\in D$, and all $a,b\in R$, we have $\operatorname{Pr}_{h \leftarrow H}[h(x) = a, h(y) = b] = \frac{1}{|R|^{2}}$. Also, *H* is an *ε-almost-strongly universal* (*ε*-ASU) family of hash functions if for any two distinct $x,y\in D$, and all $a,b\in R$, we have
  - $\operatorname{Pr}_{h \leftarrow H}[h(x) = a] = \frac{1}{|R|}$ (that is, given a randomly chosen *h* from *H*, $h(x)$ is uniformly distributed in *R*), and
  - $\operatorname{Pr}_{h \leftarrow H}[h(x) = a | h(y) = b] \leq \varepsilon $ (that is, given a randomly chosen *h* from *H*, $h(x)$ is *hard to guess* even if $h(y)$ is known).
  Equivalently, *H* is *ε*-ASU if for any two distinct $x,y\in D$, and all $a,b\in R$, we have
  - $\operatorname{Pr}_{h \leftarrow H}[h(x) = a] = \frac{1}{|R|}$, and
  - $\operatorname{Pr}_{h \leftarrow H}[h(x) = a, h(y) = b] \leq \frac{\varepsilon }{|R|}$.

Because many universal hash functions only work on fixed length messages, it is often necessary to extend the domain of the hash function to work on longer messages. Wegman and Carter [8] introduced a construction which recursively hashes messages to a desired length. Let *H* be an *ε*-AU family of hash functions, which maps blocks of length 2*l* to blocks of length *l*. At each round of tree hash, the message is split into blocks of length 2*l* and each block is hashed with some $h \in H$. The length of the message is halved each round, so the runtime is logarithmic in the size of message, and after *n* rounds of tree hash, the collision probability is $1 - (1-\varepsilon )^{n}$ [61]. However, due to the recursive nature of tree hash, it is not suitable for devices with limited memory. Instead, an iterative method can be constructed by composing hash functions.

### Theorem 3.2

([59])

*For* $i=1,2$, *let* $H_{i} : A_{i} \rightarrow B_{i}$ *be almost*-*universal families of hash functions where* $B_{1} = A_{2}$, *and define* $H = \{ h(m) = h_{2}(h_{1}(m)) | h_{1} \in H_{1}, h_{2} \in H_{2} \}$. *Then* *H* *has the following property*:

- *If* $H_{1}$ *is* $\varepsilon _{1}$-*AU and* $H_{2}$ *is* $\varepsilon _{2}$-*AU*, *then* *H* *is* $(\varepsilon _{1} + \varepsilon _{2} - \varepsilon _{1} \varepsilon _{2})$-*AU*.
- *If* $H_{1}$ *is* $\varepsilon _{1}$-*AU and* $H_{2}$ *is* $\varepsilon _{2}$-*A*Δ*U*, *then* *H* *is* $(\varepsilon _{1} + \varepsilon _{2} - \varepsilon _{1} \varepsilon _{2})$-*A*Δ*U*.
- *If* $H_{1}$ *is* $\varepsilon _{1}$-*AU and* $H_{2}$ *is* $\varepsilon _{2}$-*ASU*, *then* *H* *is* $(\varepsilon _{1} + \varepsilon _{2} - \varepsilon _{1} \varepsilon _{2})$-*ASU*.

The last two parts of this result can be used to pair an efficient *ε*-AU hash family with an *ε*-AΔU or *ε*-ASU hash family to create an efficient *ε*-AΔU or *ε*-ASU family. We can also use this result to create a Merkle–Damgård like paradigm for universal hash functions. Let $H : A \times B \rightarrow B$ be *ε*-AU and let $b \in B$. Then the family $H^{l} = \{ h_{l}(m_{l},h_{l-1}(\dots h_{2}(m_{2},h_{1}(m_{1},b)) \dots ) | h_{1},\dots ,h_{l} \in H \}$ can hash messages of length *l* for any positive *l*, and is $l(2\varepsilon - \varepsilon ^{2})$-AU. This construction was used by Minematsu and Tsunoo [62], and a more general proof on its collision bound was given by Duval and Leurent [63].

Often the collision probability of a hash function may be larger than desired. For this reason, there are several techniques for reducing the collision probability of a hash function family. If *H* is an *ε*-AU family of hash functions, then by hashing a message with two independent keys and concatenating the results, the probability of collision is lowered to $\varepsilon ^{2}$, at the expense of doubling the computational work, the length of the hash value, and the size of the key. The well-known Toeplitz extension, which has been used in several MAC algorithms (c.f. [56, 64]), reduces the key size needed for this technique. Rather than generating independent keys $\mathbf{x} = \langle x_{1}, x_{2}, \ldots , x_{k} \rangle $, $\mathbf{x'}= \langle x'_{1}, x'_{2}, \ldots , x'_{k} \rangle $, we generate the values $x_{1},\dots ,x_{k+1}$ and use the keys $\mathbf{x_{1}} = \langle x_{1}, x_{2}, \ldots , x_{k} \rangle $ and $\mathbf{x_{2}} = \langle x_{2}, x_{3}, \ldots , x_{k+1} \rangle $. We can easily extend this procedure to concatenate *n* hash values to get a collision probability of $\varepsilon ^{n}$. While the computation and hash length still increase by a factor of *n*, the size of the key only increases by *n* values. Not only does this save key material, it reduces memory accesses, thus potentially improving performance.

Now, we prove a general result for transforming *ε*-AΔU families to *ε*-ASU families. Because for authentication in QKD systems we need efficient *ε*-ASU families, our result implies that constructing such families boils down to constructing efficient *ε*-AΔU families. Our result is a generalization of the following result by Etzel *et al.* [65] which seems to have remained underappreciated.

### Theorem 3.3

*Let the family*

$$ H = \{ h_{k} : D \rightarrow R | k \in K \}$$

*be a* Δ-*universal family of hash functions*, *where* *K* *is the key space and* *R* *is a finite additive Abelian group*. *Then the family*

$$ H' = \bigl\{ h'_{k,w} : D \rightarrow R | k \in K, w \in R \bigr\} ,$$

*where*

$$ h'_{k,w}(x) = h_{k}(x) +w,$$

*and ‘*+*’ denotes the group addition operation*, *is strongly universal*.

In order to generalize the above result, we also need the following result (see [66]):

### Theorem 3.4

*Let* *G* *be an Abelian group*, *and let* $\xi _{1}, \xi _{2}, \ldots , \xi _{t}$ *be independent random variables which take on values in* *G*. *If one of* $\xi _{i}$ *is uniformly distributed in* *G*, *then the sum* $\xi _{1}+ \xi _{2}+ \cdots + \xi _{t}$ *is also uniformly distributed in* *G*.

More generally, Sherstnev [66] gave necessary and sufficient conditions on the distributions of independent random variables $\xi _{1}, \xi _{2}, \ldots , \xi _{t}$, taking on values in an Abelian group *G*, under which the sum $\xi _{1}+ \xi _{2}+ \cdots + \xi _{t}$ is uniformly distributed in *G*.

Now, we are ready to prove our result.

### Theorem 3.5

*Let the family*

$$ H = \{ h_{k} : D \rightarrow R | k \in K \}$$

*be an* *ε*-*almost*-Δ-*universal family of hash functions*, *where* *K* *is the key space and* *R* *is a finite additive Abelian group*. *Then the family*

$$ H' = \bigl\{ h'_{k,w} : D \rightarrow R | k \in K, w \in R \bigr\} ,$$

*where*

$$ h'_{k,w}(x) = h_{k}(x) + w,$$

*and ‘*+*’ denotes the group addition operation*, *is* *ε*-*almost*-*strongly universal*.

### Proof

For any two distinct $x,y\in D$, and all $a,b\in R$, we have

$$\begin{aligned} &\operatorname{Pr}_{h'_{k,w} \leftarrow H'}\bigl[h'_{k,w}(x) = a, h'_{k,w}(y) = b\bigr] \\ &\quad = \operatorname{Pr}_{h'_{k,w} \leftarrow H'}\bigl[h_{k}(x) + w = a, h_{k}(y) + w = b\bigr] \\ &\quad = \operatorname{Pr}_{h'_{k,w} \leftarrow H'}\bigl[h_{k}(x) - h_{k}(y) = a-b, w = a-h_{k}(x)\bigr] \\ &\quad = \operatorname{Pr}_{h'_{k,w} \leftarrow H'}\bigl[h_{k}(x) - h_{k}(y) = a-b \bigr] \cdot \operatorname{Pr}_{h'_{k,w} \leftarrow H'}\bigl[a = w+h_{k}(x)\bigr]. \end{aligned}$$

Since *H* is *ε*-almost-Δ-universal, we have

$$ \operatorname{Pr}_{h'_{k,w} \leftarrow H'}\bigl[h_{k}(x) - h_{k}(y) = a-b \bigr] \leq \varepsilon . $$

Also, by Theorem 3.4 we have

$$ \operatorname{Pr}_{h'_{k,w} \leftarrow H'}\bigl[a = w+h_{k}(x)\bigr]=\frac{1}{ \vert R \vert }. $$

Consequently,

$$ \operatorname{Pr}_{h'_{k,w} \leftarrow H'}\bigl[h'_{k,w}(x) = a, h'_{k,w}(y) = b\bigr] \leq \frac{\varepsilon }{ \vert R \vert }. $$

Hence, the result follows. □

## Polynomial Hash and its variants

An *ε*-AΔU family of hash functions which has received much attention is *Polynomial Hash* (PH), which is used for hashing variable length messages. The idea is that we put the message blocks as the coefficients of a polynomial and then evaluate the polynomial at the secret key, where all operations are done in a specific field or ring. In this section, we introduce and analyze several efficient variants of Polynomial Hash and then compare our results with available results. As Polynomial Hash has found many applications, our constructions and results might be of interest in various areas.

### Five variants

Here we introduce five variants of Polynomial Hash (other variants are also possible depending on applications) and analyze their universality using results from Sect. 2.

*Polynomial Hash Over Ring of Integers Modulo* *n* *(PH-IM)*: In this family, message blocks $m_{i}$ are all in $\mathbb{Z}_{p_{1}}$, where $p_{1}$ is the smallest prime divisor of *n*, the key *x* is in $\mathbb{Z}_{n}$, and all operations are performed in $\mathbb{Z}_{n}$. Formally,

#### Definition 4.1

(PH-IM)

Given an integer $n>1$ with the smallest prime divisor $p_{1}$, we define

$$ \text{{PH-IM}} := \bigl\{ h_{x} : \mathbb{Z}_{p_{1}}^{d+1} \rightarrow \mathbb{Z}_{n} | x \in \mathbb{Z}_{n}\bigr\} , $$

where

$$ h_{x}(\textbf{m}) := \sum_{i=0}^{d} m_{i}x^{i} \ (\operatorname {mod} n), $$

for every message $\mathbf{m} = \langle m_{0}, m_{1}, \dots , m_{d} \rangle \in \mathbb{Z}_{p_{1}}^{d+1}$ and every key $x \in \mathbb{Z}_{n}$.

#### Theorem 4.2

*Let* $n>1$ *has the prime factorization* $n= \prod_{i=1}^{r}p_{i}^{\alpha _{i}}$, *where* $p_{i}$*’s are prime and* $\alpha _{i} \geq 1$ *for all* *i*. *Then we have the following cases*:

- *The family PH*-*IM is* $\frac{d^{r}}{\prod_{i=1}^{r}\alpha _{i}(p_{i}-1)}$-*almost*-Δ-*universal*.
- *If* *n* *is square*-*free*, *then the family PH*-*IM is* $\frac{d^{r}}{n}$-*almost*-Δ-*universal*.
- *If* $d \geq 2$ *and* $p_{i} \geq d^{1+1/(d-1)}$ *for all* *i*, *then the family PH*-*IM is* $\frac{1}{\prod_{i=1}^{r} p_{i}^{\alpha _{i}/d}}$-*almost*-Δ-*universal*.

#### Proof

We only prove the last part; the proofs for other parts are similar. Let $\mathbf{m}=\langle m_{0}, m_{1}, \ldots , m_{d} \rangle \in \mathbb{Z}_{p_{1}}^{d+1}$ and $\mathbf{m}'= \langle m'_{0}, m'_{1}, \ldots , m'_{d} \rangle \in \mathbb{Z}_{p_{1}}^{d+1}$ be any two distinct messages. Put $\mathbf{a}=\langle a_{0}, a_{1},\dots ,a_{d} \rangle = \mathbf{m}- \mathbf{m}'$. For every $b\in \mathbb{Z}_{n}$ we have

$$\begin{aligned} h_{x}(\mathbf{m})-h_{x}\bigl(\mathbf{m'} \bigr)=b \quad \Longleftrightarrow \quad \sum_{i=0}^{d} a_{i}x^{i} \equiv b \ (\operatorname {mod} n). \end{aligned}$$

Since $\mathbf{m}\neq\mathbf{m}'$, there exists some $i_{0}$ such that $a_{i_{0}} \neq 0$. Now, we need to find the maximum number of solutions of the above polynomial congruence over all choices of $\mathbf{a}=\langle a_{0},a_{1},\dots ,a_{d} \rangle \in \mathbb{Z}_{p_{1}}^{d+1} \setminus \lbrace \mathbf{0} \rbrace $ and $b\in \mathbb{Z}_{n}$. Note that since $a_{i}$’s are in $\mathbb{Z}_{p_{1}}$ and at least one of them is not zero, we have $\gcd (a_{0}, a_{1}, \ldots , a_{d}, n)=1$. Now, by Theorem 2.14, if $d \geq 2$ and $p_{i} \geq d^{1+1/(d-1)}$ for all *i*, then the polynomial congruence

$$ \sum_{i=0}^{d} a_{i}x^{i} \equiv b \ (\operatorname {mod} n), $$

has at most

$$ \frac{n}{\prod_{i=1}^{r} p_{i}^{\alpha _{i}/d}} $$

solutions. Consequently, for part (iii) we have

$$\begin{aligned} \operatorname{Pr}_{h_{x} \leftarrow \text{PH-IM}}\bigl[h_{x}(\mathbf{m})-h_{x} \bigl( \mathbf{m'}\bigr)=b\bigr] \leq \frac{n}{n\prod_{i=1}^{r} p_{i}^{\alpha _{i}/d}}= \frac{1}{\prod_{i=1}^{r} p_{i}^{\alpha _{i}/d}}. \end{aligned}$$

 □

*Polynomial Hash With Probability At Least* $1/2$ *Zero Collision (PH-ZC)*: Let *p* be an odd prime and *k* be a positive even integer not divisible by *p*. Denote the set of even elements of $\mathbb{Z}_{p}$ by *E* and the set of odd elements of $\mathbb{Z}_{p}$ by *O*. In this family, message blocks $m_{i}$ are all in *E* or are all in *O*, the key *x* is in $\mathbb{Z}_{kp}$, and all operations are performed in $\mathbb{Z}_{kp}$. We define the family over *E*; the definition and the result over *O* are similar. Formally,

#### Definition 4.3

(PH-ZC)

Given an odd prime *p* and a positive even integer *k* not divisible by *p*, we define

$$ \text{{PH-ZC}} := \bigl\{ h_{x} : E^{d+1} \rightarrow \mathbb{Z}_{kp} | x \in \mathbb{Z}_{kp}\bigr\} , $$

where

$$ h_{x}(\textbf{m}) := \sum_{i=0}^{d} m_{i}x^{i} \ (\operatorname {mod} kp), $$

for every message $\mathbf{m} = \langle m_{0}, m_{1}, \dots , m_{d} \rangle \in E^{d+1}$ and every key $x \in \mathbb{Z}_{kp}$.

#### Theorem 4.4

*The family PH*-*ZC is* $\frac{d}{p}$-*almost*-Δ-*universal*. *Furthermore*, *with probability at least* $1/2$ *the collision probability is exactly zero*.

#### Proof

Let $\mathbf{m}=\langle m_{0}, m_{1}, \ldots , m_{d} \rangle \in E^{d+1}$ and $\mathbf{m}'= \langle m'_{0}, m'_{1}, \ldots , m'_{d} \rangle \in E^{d+1}$ be any two distinct messages. Put $\mathbf{a}=\langle a_{0}, a_{1},\dots ,a_{d} \rangle = \mathbf{m}- \mathbf{m}'$. For every $b\in \mathbb{Z}_{kp}$ we have

$$\begin{aligned} h_{x}(\mathbf{m})-h_{x}\bigl(\mathbf{m'} \bigr)=b \quad \Longleftrightarrow \quad \sum_{i=0}^{d} a_{i}x^{i} \equiv b \ (\operatorname {mod} kp). \end{aligned}$$

Since $\mathbf{m}\neq\mathbf{m}'$, there exists some $i_{0}$ such that $a_{i_{0}} \neq 0$. Now, we need to find the maximum number of solutions of the above polynomial congruence over all choices of $\mathbf{a}=\langle a_{0},a_{1},\dots ,a_{d} \rangle \in E^{d+1} \setminus \lbrace \mathbf{0} \rbrace $ and $b\in \mathbb{Z}_{kp}$. Since $m_{i}$’s are all even, $a_{i}$’s are also all even. For every $b\in \mathbb{Z}_{kp}$, if *b* is odd then the polynomial congruence

$$ \sum_{i=0}^{d} a_{i}x^{i} \equiv b \ (\operatorname {mod} k), $$

has no solution, but if *b* is even then it has at most *k* solutions. On the other hand, by Lagrange’s Theorem, for every $b\in \mathbb{Z}_{kp}$ the polynomial congruence

$$ \sum_{i=0}^{d} a_{i}x^{i} \equiv b \ (\operatorname {mod} p), $$

has at most *d* solutions. Now, using Theorem 2.12 the polynomial congruence

$$ \sum_{i=0}^{d} a_{i}x^{i} \equiv b \ (\operatorname {mod} kp), $$

has no solution if *b* is odd, and has at most *kd* solutions if *b* is even.

Consequently, we have

$$\begin{aligned} \operatorname{Pr}_{h_{x} \leftarrow \text{PH-ZC}}\bigl[h_{x}(\mathbf{m})-h_{x} \bigl( \mathbf{m'}\bigr)=b\bigr] \leq \frac{kd}{kp}= \frac{d}{p}. \end{aligned}$$

Note that although $\frac{d}{p}$ is an upper bound for the collision probability, but when *b* is odd and possibly in other cases (so with probability at least $1/2$) the collision probability is exactly zero. Hence, the result follows. □

*Polynomial Hash Over Prime Fields (PH-PF)*: In this family, each message block $m_{i}$ and the key *x* are in $\mathbb{Z}_{p}$, and all operations are performed in $\mathbb{Z}_{p}$. Formally,

#### Definition 4.5

(PH-PF)

Given a prime *p*,

$$ \text{{PH-PF}} := \bigl\{ h_{x} : \mathbb{Z}_{p}^{d+1} \rightarrow \mathbb{Z}_{p} | x \in \mathbb{Z}_{p}\bigr\} , $$

where

$$ h_{x}(\textbf{m}) := \sum_{i=0}^{d} m_{i}x^{i} \ (\operatorname {mod} p), $$

for every message $\mathbf{m} = \langle m_{0}, m_{1}, \dots , m_{d} \rangle \in \mathbb{Z}_{p}^{d+1}$ and every key $x \in \mathbb{Z}_{p}$.

#### Theorem 4.6

*The family PH*-*PF is* $\frac{d}{p}$-*almost*-Δ-*universal*.

#### Proof

Same as above, just use Lagrange’s Theorem or Theorem 2.4. □

#### Remark 4.7

It is important to note that we do not have to restrict the message blocks to be in $\mathbb{Z}_{p}$ or $\mathbb{Z}_{n}$. In fact, the message blocks can be *arbitrary* non-negative integers as long as no two messages have all their corresponding blocks congruent modulo *p* or modulo *n*. See Theorem 4.9 for an example of such constructions in the case of $\mathbb{Z}_{p}$ but the same technique is also applicable to $\mathbb{Z}_{n}$.

*Polynomial Hash Over Prime Fields With Arbitrary Message Blocks (PH-PA)*: Let *A* be a subset of $\mathbb{Z}_{\geq 0}^{d+1}$ such that no two elements of *A* have all their corresponding coordinates congruent modulo *p*. In this family, each message **m** is in *A*, the key *x* is in $\mathbb{Z}_{p}$, and all operations are performed in $\mathbb{Z}_{p}$. Formally,

#### Definition 4.8

(PH-PA)

Given a prime *p*,

$$ \text{{PH-PA}} := \{h_{x} : A \rightarrow \mathbb{Z}_{p} | x \in \mathbb{Z}_{p}\}, $$

where

$$ h_{x}(\textbf{m}) := \sum_{i=0}^{d} m_{i}x^{i} \ (\operatorname {mod} p), $$

for every message $\mathbf{m} = \langle m_{0}, m_{1}, \dots , m_{d} \rangle \in A$ and every key $x \in \mathbb{Z}_{p}$.

#### Theorem 4.9

*The family PH*-*PA is* $\frac{d}{p}$-*almost*-Δ-*universal*.

#### Proof

Same as above, just use Lagrange’s Theorem. Note that when we find the difference of the two polynomials, at least one of the coefficients is non-zero modulo *p* (by the definition of the set *A*) so the assumption of Lagrange’s Theorem is satisfied. Also, note that Theorem 2.4 is not applicable here because message blocks are not necessarily in $\mathbb{Z}_{p}$. □

*Polynomial Hash Over Finite Fields (PH-FF)*: In this family, each message block $m_{i}$ and the key *x* are in $\mathbb{F}_{q}$, and all operations are performed in $\mathbb{F}_{q}$. Formally,

#### Definition 4.10

(PH-FF)

Given the finite field $\mathbb{F}_{q}$ with *q* elements, where *q* is a prime power,

$$ \text{{PH-FF}} := \bigl\{ h_{x} : \mathbb{F}_{q}^{d+1} \rightarrow \mathbb{F}_{q} | x \in \mathbb{F}_{q}\bigr\} , $$

where

$$ h_{x}(\textbf{m}) := \sum_{i=0}^{d} m_{i}x^{i}, $$

for every message $\mathbf{m} = \langle m_{0}, m_{1}, \dots , m_{d} \rangle \in \mathbb{F}_{q}^{d+1}$ and every key $x \in \mathbb{F}_{q}$.

#### Theorem 4.11

*The family PH*-*FF is* $\frac{d}{q}$-*almost*-Δ-*universal*.

#### Proof

Same as above, just use Theorem 2.4. □

#### Corollary 4.12

*Using Theorem *3.5, *any* *ε*-AΔ*U family*, *in particular the families studied in this paper*, *can be transformed to* *ε*-*ASU families which makes them useful for various applications including authentication of variable length messages in QKD*. *This can be done by adding a uniform value* $w \leftarrow R$ *to the hash functions*, *where* *R* *is the range of the corresponding hash functions*.

### Comparison and remarks

The above techniques and results on the Polynomial Hash and its variants and comparing them with what were known before, reveals some remarks:

- Polynomial Hash is widely attributed to Wegman and Carter [8], Dietzfelbinger et. al. [67], den Boer [68], Bierbrauer et. al. [69], and Taylor [70]. But we have discovered that it has been already introduced by Mehlhorn and Vishkin [71] back in 1984 (of course, Wegman and Carter [8] already studied the degree one case).
- So far, only the families PH-PF and PH-FF have been introduced in the literature but, unfortunately, there is a growing number of papers that explicitly or implicitly have used the Fundamental Theorem of Algebra to prove the *ε*-almost-Δ-universality of these families. As discussed in detail in Remark 2.5, the Fundamental Theorem of Algebra works only over the field of complex numbers not over the prime field or finite fields, so is not applicable to the families PH-PF and PH-FF. Instead, those papers should have used Lagrange’s Theorem or Theorem 2.4 as we did.
- Polynomial Hash has been already used to provide a very efficient universal hash function family, for authentication in QKD [22–24] but it has not been explained why that is the case. In fact, the efficiency of Polynomial Hash comes from at least the following observations:
  - The evaluation of a polynomial $f(x)$ of degree *d*,
    $$ f(x)=a_{d}x^{d} + \cdots + a_{1}x + a_{0} $$
    needs only *d* multiplications and *d* additions since, by Horner’s rule, $f(x)$ can be written as
    $$ f(x)= a_{0} + x \bigl(a_{1} + x \bigl(a_{2} + x \bigl(a_{3} + \cdots + x(a_{d-1} + x a_{d}) \cdots \bigr) \bigr) \bigr). $$
    Therefore, hashing a message of length $d+1$ using Polynomial Hash needs only *d* multiplications and *d* additions, while hashing the same message using most other universal hash function families needs more computations, for example, hashing it using MMH^∗^ [56, 72, 73] (which is one of the most well-known universal hash function families) needs $d+1$ multiplications and $d+1$ additions.
  - Unlike most other universal hash function families (e.g., MMH^∗^ and its variants) which hash fixed length messages (that is, once the key is chosen we can only hash message of the same length as the key) Polynomial Hash can be used for hashing variable length messages because each message block becomes the coefficient of the polynomial, and so is independent of the key.
- Even though the collision bounds of the hash families introduced in this paper are quite strong, even if for some application we pick a family with a slightly weaker collision bound thanks to the *everlasting security* of QKD [5, 6, 74] if authentication remains unbroken during the execution of the QKD protocol, then the resulting key is information-theoretically secure; breaking authentication after the protocol has output the key will not change the security of the generated key.
- As universal hashing is used not only for authentication in QKD but also in other steps in QKD like error correction and privacy amplification [7, 13, 25–30], our constructions and results might lead to improvements in QKD protocols, among other areas.
- Universal hash functions have been recently used in studying quantum secure direct communication (QSDC) [75] (see also, [76–80]), quantum secret sharing (QSS) (either directly [81, 82] or via a security proof based on QKD [83]), quantum conference key agreement (QCKA) [84–86], and quantum authentication [87–89]. Therefore, our efficient and secure constructions and results might lead to improvements in these directions as well.
- Our study of Polynomial Hash over $\mathbb{Z}_{n}$ and its variants also demonstrate various benefits which do not hold in the case of the two well-known variants of Polynomial Hash. In particular,
  - We do not have to restrict the message blocks to be in $\mathbb{Z}_{p}$ or $\mathbb{Z}_{n}$. In fact, the message blocks can be *arbitrary* non-negative integers (unlike the two well-known versions). See Remark 4.7 and Theorem 4.9 for the details.
  - In some of these variants with probability at least $1/2$ the collision probability is exactly zero (see Theorem 4.4).
  - We do not need large prime numbers or finite field arithmetic anymore (that is, all arithmetic is done in $\mathbb{Z}_{n}$).
  - It is also possible to introduce, generalize, and analyze other variants of Polynomial Hash (for specific applications) using results from Sect. 2.
  - Although in QKD the legitimate parties need to share some initial small secret information in advance for the authentication of the classical channel, each round of QKD provides substantially larger fresh key materials, part of which can be used for authentication in the next round of QKD. Furthermore, keys generated in each round of QKD are completely independent of all prior keys and messages [5, 6, 74]. Therefore, even if any of our schemes uses more key materials at the expense of other benefits, the protocol compensates it in the next round.
  - We connected Polynomial Hash and QKD with deep results in number theory. This may motivate more work in these areas.

## Data Availability

No data is needed for this work.
